# Drilling for ‘New Oil’ in Care Integration – Co-Production of the Concept and Specification of an Integrated Data Centre for Policy Decision Making, Care Planning, and Research in Estonia

**DOI:** 10.5334/ijic.6953

**Published:** 2023-03-29

**Authors:** Ingo Meyer, Gerli Aavik-Märtmaa, Adriana Poppe, Sonja Müller, Leo Lewis, Andrew Terris, Doris Kaljuste, Marion Rummo, Kadri Rootalu, Marko Bucik

**Affiliations:** 1 PMV research group, Medical Faculty and University Hospital Cologne, University of Cologne, Germany; 2 Tallinn University of Technology, Tallinn, Estonia; 3 empirica Gesellschaft für Technologie- und Kommunikationsforschung mBH, Germany; 4 International Foundation for Integrated Care, UK; 5 Estonian Ministry for Social Affairs, Estonia; 6 Statistics Estonia, Estonia; 7 European Commission, UK

**Keywords:** data integration, evidence-based decision making, population health, data centre, integrated care

## Abstract

**Introduction:**

Care integration needs to take place on different levels, including that of infrastructure and especially data infrastructure. Only integrated data allow for policy making, care planning, research, and evaluation that spans across different sectors of care and support.

**Methods:**

In the course of an EU-funded reform initiative on integrated care, the Estonian government and various agencies have developed a concept for an integrated data centre, bringing together information from social, medical, and vocational support services. The concept was developed in co-production with many stakeholders. A test data set from all covered sectors, including the pseudonymised data of 17,945 citizens of an Estonian municipality, was created and analysed as a proof-of-concept exercise.

**Results:**

The co-production approach resulted in a set of requirements and use cases as well as a specification of premises, processes, and data flows for the data centre. The analysis of the test dataset showed the principal feasibility of the dataset for the intended purposes.

**Conclusion:**

The concept development phase showed that an integrated data centre for Estonia is feasible per se and helped to specify concrete actions required for its realisation. Strategic and financial decisions from the Estonian Reform Steering Committee are now needed to create the data centre.

## Introduction

“The world’s most valuable resource is no longer oil, but data”, headlined The Economist in 2017 [1]. This catchphrase has been on everyone’s lips for some time now. In healthcare, this debate is much older. Whether it is evidence-based medicine at the level of the individual [2] or evidence-based health policy making on the level of populations [3]: the use of different types of evidence – from clinical studies, administrative data, real-world data and other sources – is an essential part of creating and maintaining health and wellbeing. New approaches, such as learning health systems, are being used to systematically gather, create and apply evidence in care settings [4]. However, challenges remain. A major problem in achieving person-centred and integrated care is not only the fragmentation of care delivery across healthcare, social services and benefits, unemployment assistance, education, and other areas but also the fragmentation of governance structures, financing, and information processing, i.e., the very basis for the extraction of evidence. In particular, the fragmentation of information systems contributes strongly to a situation where no agreement can be formed across different sectors about what data already exists that can be a proxy for integration and how it can become an operational and learning dataset. In other words, information systems need to be integrated just as much as the service level to provide integrated care [5].

Estonia is attempting to overcome the challenges of fragmented health and care systems. The central long-term strategy for the Estonian government, titled “Estonia 2035”, highlights integrating health and social services in a person-centred way as one of its aims for the next 15 years. The goal is to move towards services that operate in the background of people’s life paths and are supported by digital solutions and integrated use of data [6]. Estonia is also highly advanced when it comes to digital solutions [7]. This includes the health sector: the Bertelsmann Foundation’s Digital Health Index ranked Estonia 1^st^ in 2019, including in the category of actual use of data [8]. Estonian e-health solutions and services, such as electronic health records and e-prescriptions, are advanced, but improvements can still be made. Among other things, in using data for enabling service integration [9]. People with more complex needs often simultaneously need services from health, welfare and educational or employment sectors. Despite efforts to better align the services, the overall system is fragmented and not all registries are able to systematically exchange data. This results in an unnecessarily high number of assessments, applications and other paperwork that people with complex needs are responsible for completing [10]. The Burden of Care Task Force, a strategic initiative aiming to map and work out solutions for reducing the high burden of care in Estonia, identified the need for better integrated care systems already in 2017. Lack of integrated use of data was seen as one of the key factors hindering integration [11]. At that time, several pilot projects had been launched, aiming to reduce the gap between health and welfare services and improve data exchange between these sectors. However, there was no overall strategy for improving integrated care and no active attempts to establish cross-sectoral integration of data for better policy planning and analysis, though the need for it was being widely acknowledged among policymakers.

In 2018, a reform initiative was launched with the support of the Technical Support Instrument of the European Commission, aiming to overcome the project-based approach and develop a wholistic integrated care strategy. As the initiative took place in a complex multi-stakeholder environment, a co-production approach was adopted, bringing a variety of expertise and experiences together [12]. In complex systems, co-production can help balance the different levels of power held by stakeholders, enabling a variety of actors to have influence on policy processes. Co-production allows service providers to understand other perspectives, potentially leading to more efficient services and overcoming thinking and acting in departmental silos [13]. Examples of co-production are often found when previous models of public service delivery have failed – such previous attempts can then be treated as learning experiences and a ‘learning by doing’ approach can be taken in partnership with other stakeholders [14]. Also, governments need to be increasingly able to react to rapid changes, thus the “how” of policymaking is becoming just as important (if not more) as the “what” [15]. From the get-go, the most critical part of the reform initiative was cross-sectoral integration of data. This paper provides an in-depth description of the reform initiative’s data workstream and how it went from a country-wide stocktaking exercise via stakeholder consultations to developing the concept and specifications of an integrated data centre aimed to support policy decision making, care planning, and research in Estonia.

## Building an integrated data centre

### Overview of the reform initiative

The core aim of the integrated care reform initiative was to contribute to a more integrated and person-centred provision of social, medical, and vocational support services to people with disabilities and to older people with high support needs. This was to be done through the provision of new tools, processes, and capacity-building for a variety of stakeholders in Estonia, so that they could better answer questions, such as how to improve value for those in need, where to best direct limited resources, how to use data more effectively, how to measure the impact of integrated care and how to incentivise integrated care. The initiative was built around four inter-linked workstreams:

1. **Integration Strategy workstream** aiming to create a common focus and an agreed path towards the development and adoption of a high-level strategy to implement integrated care;
2. **Models of Care workstream** aiming to design processes for more streamlined care pathways, including views on information flows, roles, and financing flows. A special focus was given to the functions and competencies of care coordination that supported a care co-ordination model roll-out going on in parallel in different regions of Estonia;
3. **Data and Information workstream** aiming to design an optimal model of linking the various relevant data to support analysis for policy design and delivery of integrated care. The key elements here were the design of a test dataset that uses the available electronic data from different state and local municipality registries for policy analysis and a proof-of-concept analysis of this dataset with the data of the residents of a specific municipality in Estonia;
4. **Financing and Incentives workstream** aiming to recommend the best financing measurements and incentives for enabling integrated care.

### Methodological approach

For the overall process, the approach of a *design science* was used [16]. Similarly to the process described by vom Brocke et al. [17], the work started with a general idea of pulling together pseudonymised data from various existing registries, allowing to better understand what kind of support is being provided to different cohorts of people with similar characteristics. The co-creation process commenced with the introduction of the overall idea, which was then complemented with the help of different stakeholders. Several iterations of the initial dataset descriptions were done before starting the proof-of-concept exercise with real data. During the proof of concept exercise, additional changes were made, and finally, a concept of integrated data centre was developed. Triangulation of methods was used to increase the credibility of the work [16], combining document analysis, knowledge gathered in a series of co-production workshops, meetings and interviews with relevant national and international experts, and a proof-of-concept analysis with a test dataset combined for the purposes of this exercise.

### Co-production of the data centre

The Data and Information workstream’s co-production approach started with a stocktaking exercise, followed by requirements elicitation and proof-of-concept phases to arrive at the definition of an integrated data centre. This final outcome itself was not predefined but emerged during the initial stages of the process, based on the demand voiced by the stakeholders involved.

**Figure 1:**
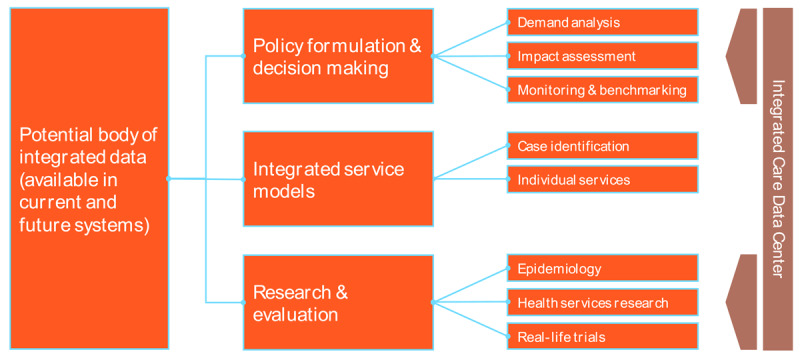
Potential purposes of an integrated data system.

The first step in the approach was to create an inventory of existing data systems in all relevant sectors of care and support, mapping the systems currently existing and the kind of data that is being collected and processed. This inventory was then compared to a normative view of what kind of data and information would be required. During the first co-production activity, this normative view was augmented by input from stakeholders representing different support sectors. It outlined the information that is currently missing and preventing the achievement of a higher degree of integration. Based on this, a potential body of integrated data cutting across several sectors of care and support could be used for three main purposes (see Figure 1 below) using data:

1. in policy and other decision-making processes to better align them with demand in different parts of the system and different geographical areas of Estonia;
2. to support integrated service delivery, including case identification and individual service provision;
3. in research & evaluation.

Further stakeholder consultations revealed that parts of this functional architecture and their related purposes were more likely to be achieved within the short- or medium-term than others. Data integration that supports individual service delivery was considered to be the most challenging objective as it requires the – more or less – real-time flow of person-identifiable data across different systems, which creates both technical and privacy concerns. Compared to this, the collation of data for the purposes of decision making, planning, and research was seen as more readily achievable, since it is possible to work with pseudonymised or even anonymised data, as well as to condone longer time lags from data delivery to analysis. Based on these outcomes, the Reform Steering Committee agreed to focus further work in the Data and Information workstream on the development of a concept for an integrated data centre that can support policy formulation, decision making, research, and evaluation on the basis of pseudonymised data that are available with a certain delay.

In the second round of co-production activities, the requirements of various stakeholders vis-à-vis such an integrated data centre were collated and implementation aspects discussed in a series of expert consultations. This involved representatives from four groups:

- representatives from the frontline service level, i.e., from municipalities, hospitals, GPs/GP networks, and social service providers (Group 1);
- state level stakeholders involved in system governance and financing (Group 2);
- technical experts with expertise in data system design, data management, and data analytics (Group 3);
- legal experts (Group 4).

Stakeholders from Groups 1 and 2 were mostly involved to elicit functional requirements, answering questions such as “What use cases should the integrated data centre be able to serve?” and “What data demands should the centre be able to satisfy?” from their perspective. Stakeholders from Group 3 were mostly involved to outline the technical requirements of how such a centre could be made a reality within the existing technical and organisational infrastructure in Estonia. The legal experts focused on legal and regulatory requirements and how they need to be taken into consideration. Additionally, consultations with Groups 3 and 4 helped avoid redundant techno-optimism, sometimes prevalent in similar exercises [18] and plan a roadmap, where first actions could be realistically developed in the short-term future.

An additional consultation was held with representatives of the German Research Data Centre for Health Data (FDZ DaTraV), which is operated by the German medical regulation body BfArM (Bundesinstitut für Arzneimittel und Medizinprodukte). Furthermore, consultation with stakeholders working with integrated datasets or data centres in Germany (CoRe-Dat), Spain (Badalona Serveis Assistencials – BSA), Australia (including representatives from the Australian Bureau of Statistics, Australian Institute of Health and Welfare, Australian Government Department of Health, and Australian Digital Health Agency) and New Zealand (including representatives from the Ministry of Health NZ, Central Region’s Technical Advisory Services, Social Wellbeing Agency, and Canterbury District Health Board) were organised. These consultations focused on gaining practical experience from the international cases that could be of relevance and value for Estonia.

#### Requirements and use cases of the data centre

The workshops resulted in several requirements for the data centre as well as an indicative set of use cases that the centre should be able to serve. Requirements fell into four categories and are presented in a summarised form below, with the full requirements and use cases presented in the annex (see section 8).

##### General requirements

General requirements formulated in the workshops included that the data centre should be set up as a socio-technical system [19], combining human, technical, organisational, and legal components. This should include elements of a learning system, allowing for a regular assessment of the performance of the data centre, a regular comparison of the demand for data and what the data centre is able to supply, as well as a continuous process for improvement. The data centre should be accessible easily and quickly. This requirement stemmed from the fact that current turn-around cycles for policy data were deemed too long, and especially procedures to obtain access to and assemble datasets were considered too complicated. The data centre should also operate in accordance with good scientific practices and relevant scientific methodological guidelines.

##### Legal and regulatory requirements

There was a consensus among legal experts (Group 4) that the data centre would require a dedicated legal framework that currently does not exist in Estonian law. The legal framework should specifically allow the data centre to collect and process pseudonymised, individual level data. All experts interviewed expressed the unambiguous view that anonymised or aggregate data are insufficient. Furthermore, the data centre should be allowed to request, collect, and process data from all relevant data owner organisations without having to go through the lengthy data protection and ethics procedures that are currently required. This concerns specifically the collection of data on the municipality level and the data from service providers. One strong advantage in the Estonian system is the usage of unique personal identification codes, which significantly simplifies the linking of individual level data.

##### Governance requirements

Experts stated that the centre would need product owners with clearly defined roles, rights and responsibilities. The centre should also be set up in a way that is organisationally separate from any agency providing data, as well as outside any form of scientific or economic competition (e.g., for research grants). Users and analysts of the data should be able to receive support from the data centre, e.g., in the form of information and training materials or training workshops.

#### Initial set of use cases

Elaborating on the general purposes of the data centre, experts developed a set of use cases for the data centre. These were considered initial use cases to be further developed when the data centre is being set up:

- identification of priority areas for further integration measures, e.g., by population size, financial impact, shortcomings of the current support structure;
- developing and testing different financing models for integrated care specific to the Estonian context, including sensitivity to high-cost cases, resilience in case of pandemics or other disasters etc.;
- facilitation of the development of clear metrics that are credible to drive, e.g., reimbursement decisions;
- understanding demand and population structures for integrated care pathways, including changes over time;
- identifying key hand-over points in existing care trajectories that would require coordination measures;
- developing and testing suitable measures for the monitoring and benchmarking of integrated care along the line of the quadruple aim [20] of 1) population health (in the sense of objective care and support outcomes), 2) the users’ perception of care outcomes and their experience of service provision, 3) the cost-effectiveness of service provision and 4) the experience of service providers [21].

A more detailed overview is given in the annex (see section 8.1.1.).

### Proof-of-concept: assembling and analysing an integrated test dataset

An integrated test dataset was assembled and analysed as a proof-of-concept, mainly for two purposes:

- to determine the general feasibility of such a linked dataset, esp. with a view to the plausibility and quality of the linked data and;
- to carry out several content-related proof-of-concept analyses to test the dataset’s potential to deliver evidence that can support policy decision making and care planning in relation to integrated care.

The test dataset combined data from different sectors for a population of people living in the municipality of Viljandi in Southern Estonia (n = 17,945). The observation period was from January 2017 to December 2018, which means that services included in the dataset should have been provided in these two years. The data were selected from the data systems of different agencies and organisations involved in providing care, support, and benefits (see Table 1 in the annex for details). This covered basic registration data (such as date of birth, gender), health services data (e.g., use of services, health diagnoses), education data (e.g., educational attainment level, educational special needs), social service data (times and results of needs assessments, use of services, etc.) and employment-related data (e.g., employment status, receipt of unemployment benefits). Data from the different systems were linked using a pseudonym based on the Estonian unique personal identification code. Most data on services, benefits, assessments, etc. came with a time stamp or a duration exact to the day, in principle allowing for an analysis of people’s trajectories across the different sectors, receipt of overlapping services and/or benefits, and similar analyses.

The test dataset was compiled by Statistics Estonia on a protected analytics server, with the study team receiving remote access to carry out the analysis itself.

#### Ethical approval

In order to carry out the proof-of-concept exercise, permissions from the Estonian Data Protection Agency and an ethics committee were sought and obtained. Throughout these processes, any possible risks and measures to be taken to mitigate such risks were described in detail. However, if the envisioned integrated dataset were to be created for ongoing policy support, more permanent and less time-consuming solutions for ensuring the integrity of data are critically needed.

#### Plausibility and quality of the linked test dataset

**Figure 2:**
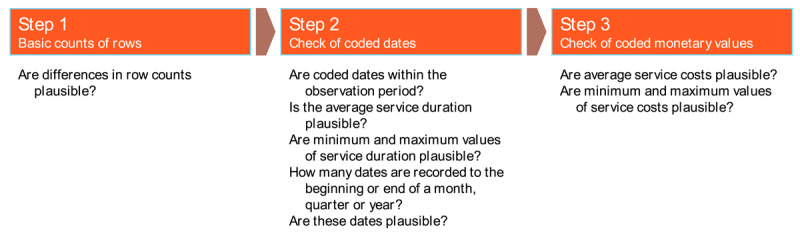
Three steps of plausibility and quality checks.

To ensure plausibility and quality, the raw data were imported into the analytic system and variable names were harmonised across data tables. Where necessary, missing data was marked up appropriately and the data was recoded to fit the variable declarations described in the minimum dataset. The analysis of data plausibility and quality was done in three steps, as shown in Figure 2.

#### Content-related proof-of-concept analyses

For the content related analysis of the test dataset, a series of research questions was formulated in a workshop with representatives from all organisations participating in the reform initiative. Most analyses in this part concerned individual service trajectories and their characteristics, starting with basic information, such as the number of services and benefits a person received and the length of service and benefit periods. More specific questions addressed the proportion of people, whose trajectory included services provided in institutional settings, such as care homes or hospitals. The last area concerned overlaps of services and benefits and whether these were plausible or permissible in principle or whether certain sequences could be seen as an indication that service objectives were not being achieved.

#### Summary of results

In summary, both the analysis of data plausibility and quality and the content-related analysis were able to show that the test dataset can, in principle, be used to fulfil the defined analytic purposes and deliver aggregated data for planning and decision-making purposes. The dataset provided a coverage of all relevant sectors of care and support and allowed bringing those together into a coherent view.

### Integrated data centre: premises, processes, and data flow

Based on the requirements analysis and the proof-of-concept analysis, the overall concept, as well as the process and data flow for the integrated data centre were developed by the core team of the reform initiative, presented to all initiative participants, and refined accordingly.

#### Premise

The concept is based on a number of premises. Firstly, it should be considered an initial version of the integrated data centre, aiming to fulfil specific functions and deliver specific data products. In line with the concept of the learning socio-technical system approach that emerged from the requirements analysis, these premises are subject to change. Second, the data centre is to correspond to two types of data products to be offered:

1. analytic reports, relating to one-off analysis and based on specific data requests and;
2. dashboards of regularly updated data on given subjects and based on specific data requirements.

Third, the data centre is not based on a permanently assembled centralised dataset, but rather on individual data requests from the delivering agencies, directly linked to each analytic task (reports of dashboards). This implies that the process of learning and continuous improvement of the data centre is limited to the methods and procedures surrounding data delivery, data preparation, quality assurance and analysis, rather than including a continuously improving dataset itself. This approach should be monitored both in terms of its effectiveness and its efficiency, depending also on the number of data requests to be handled within a specified time frame.

#### Process and data flow

**Figure 3:**
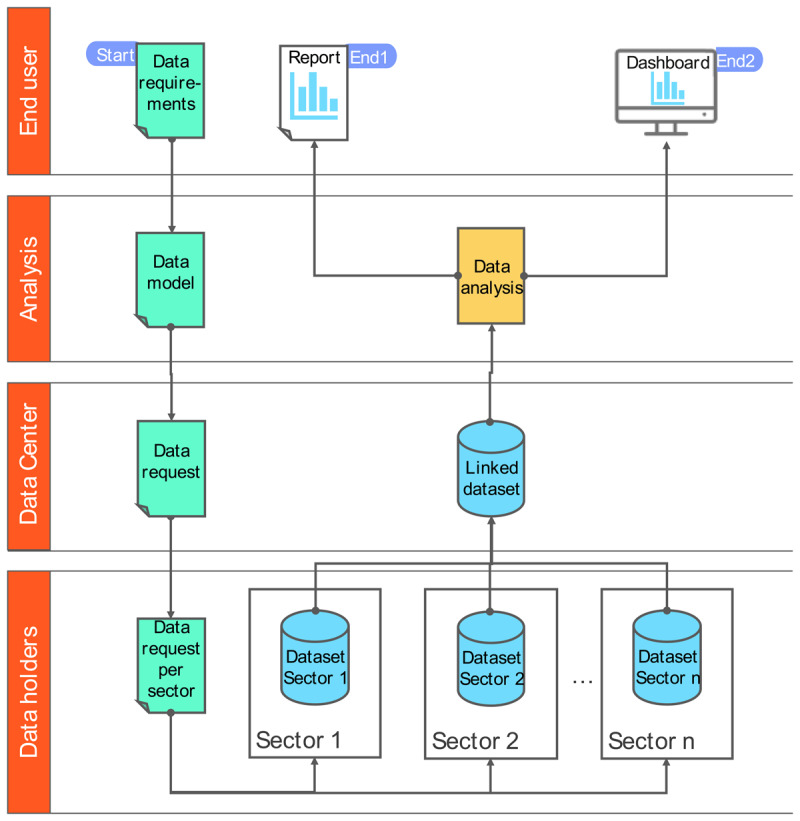
Process and data flow integrated data centre.

The process and data flow are presented in Figure 3. A full view is included in the annex in Table 2, providing more details on pseudonymisation and encryption of the data, as well as on different process steps for data staging carried out within the data centre.

The process starts with the end-user and is identical for a) data products, b) a one-off data request for a report or, c) a request for data for a continuous dashboard. The specific data requirements for the data product are specified by the end-user. These requirements are then translated into a specific data model by an analyst. This data model needs to comply with the overall structure of the available data. To support this, the data centre may provide a description of all available data profiles, referencing types of data, data fields, data formats, etc. The data model also contains justifications for all data being requested that are specifically linked to the analytic purpose to be served by the data product. The data model is transferred into a data request split into different sectors or data holders from which data is being requested. These sector-specific data requests are transmitted to the data holders by the data centre. Each data holder selects data based on the data request from its own source system or systems. The resulting datasets are delivered to the data centre via secure means, and record linkage and quality assurance are carried out. The linked dataset is made available to the analysts for data analyses. Depending on the specified product, either an analytic report or a dashboard input is produced.

Based on the requirements established in expert workshops, two alternative approaches for the data centre were also considered. The first alternative was to maintain a permanent general dataset at the data centre rather than assemble one for each request. Another alternative approach would be to not carry out the analysis on an assembled dataset (persistent or one-off), but in different source systems with only results being handed back to the analysts (“algorithm to the data”). However, the approach described in previous sections was considered the most suitable.

## Discussion

### Comparison with similar activities across the world

The Estonian initiative for an integrated data centre needs to be seen in the context of a global push towards care integration that encompasses data integration [5,22]. Different approaches have been developed around the world that are to some degree comparable to the approach presented here. Several of those, namely from Germany, Spain, Australia, and New Zealand, were consulted with in the course of this reform, with the examples from New Zealand and Australia being the most relatable, as both have country-wide coverage and data going beyond the healthcare sector. The most important lessons to draw from these cases concerned the governance of the data. In both contexts, objectives and values of any data linkage work were well defined and described, significant investments had been made to ensure that key stakeholders were equipped with the skills needed for the use of linked data, proper and transparent management and governance system were set up and people with dedicated time and resources were working on building and maintaining the datasets. The regional examples of CoRe-Net (Cologne) (18) and BSA (Badalona) were examined in more technical detail, but comparisons were possible only to some extent, as the Estonian initiative has a broader coverage in terms of the sectors of care and support that are to be included as data holders. The Estonian concept also allows for a full coverage of the country’s population, which again seems only to be achieved in the Australian and New Zealand examples.

### Strengths and weaknesses of the planned data centre

This section discusses the planned dataset and data centre, relying on the proof-of-concept dataset exercise, stakeholder discussions, and experiences from other contexts. The main focus will be on the dataset, with some reflections on the overall data centre concept and the Estonian context. In the course of the exercise, a variety of (potential) strengths and weaknesses were identified.

Mcbride et al. [23] have described six factors of a ‘perfect storm’ for a data-driven co-creation, five of which can also be seen in the Estonian example: motivated stakeholders (able to see the potential gain for their organisations), innovative leaders (willing to think outside the borders of their own sectors and organisations), proper communications (co-production approach ensuring the commitment of relevant stakeholders), external funding (financial support from the European Commission), and agile development (learning by doing approach taken in all steps). The presence of these factors in the Estonian case is creating good prerequisites for success. The only factor not present is the existence of an open government portal containing the data under question. This is a reflexion on the overall (open) data availability and data reuse. While Estonia ranks high in several digital governance indexes (e.g., first in Digital Economy and Society Index 2022 [24] and UN e-Government Survey Online Services Index 2022 [25], the OECD OURdata Index 2019 shows that Estonia is one of the lowest performing countries with regards to government support for data reuse and below OECD average in the overall ranking [26]. In terms of strengths, the data centre is based on a broad integration concept, including both local and national level information and pulling data from health, welfare, employment, and education sectors. This permits answering a wide array of questions around integrated provision of care and support. As those questions were developed in close collaboration with stakeholders from different agencies in Estonia, they can be said to represent real issues that those agencies are faced with and are unable to address with the use of current (segmented) information systems. Furthermore, the proof-of-concept analysis showed that it is possible to answer a range of different questions on the basis of the data. The dataset is in principle capable of covering the full population of the country, with an opportunity to focus on a municipality or a region, while being based solely on administrative, secondary data. This view is – again in principle – not subject to the biases that impact primarily collected data, in particular selection biases such as response bias or loss to follow-up. Due to the use of pseudonymised data, all data can be collected from source systems and analysed without individual consent. As a result of the exclusive use of secondary data, data can be obtained with comparative ease and at comparatively low cost when compared to primary data collection exercises. Finally, the data privacy footprint of the dataset is comparatively low due to the use of pseudonymised data and the applied data flow with pseudonymisation at the source. The measures used in addition to pseudonymisation are described in the annex under section 8.1.2.

Overall, the establishment of a data centre, capable of pulling and analysing integrated datasets, could provide a data-based starting point for problem solving for both local and national level policymakers, thus increasing the capabilities of the ministries, agencies, and local municipalities alike. As the data centre would have relevant know-how and equipment ready for pulling and analysing needed datasets and would either have legal grounds for its work or smooth ethics review processes at place, the preparation time for necessary datasets could be significantly reduced.

The main weakness of the dataset relates to it being based on secondary data that was originally collected for administrative purpose. This means that part of the data is subject to administrative prevalence, which can be seen as a type of imbalance in needs and prone to underestimating the match of supply and demand. Administrative prevalence impacts the data in two ways. First, the dataset only contains data on people who came into contact with the system at some stage. Second, data about an individual appears in the dataset without providing a complete picture of that individual’s situation, because additional data were not required for the administrative purpose.

Another major weakness is the general lack of coverage of certain topics, which is also related to the administrative nature of the data. A common normative view of the goals of care integration revolved around the so-called quadruple aim [20] and it is commonly understood that outcomes along all four dimensions need to be balanced in order to achieve a working and sustainable integrated care scheme. By inference, a dataset aiming to support decision making for integrated care should cover all the elements of the quadruple aim, at least to some extent. The test dataset assembled here does this only to a limited degree, especially in relation to population health and cost. The user and service provider experiences however are missing from the dataset. On the other hand, the creation of the integrated data centre has the potential to improve data collection and quality through feedback to data owner organisations. Also, while the pseudonymised cohort data has major potential to improve policy formulation and decision making, prioritising only that purpose, means that individual cases still cannot be identified to organise needed care.

While the overall digitalisation level in Estonia is very high, there are several issues that need to be addressed when a real-life data system would be put into place. The most fundamental of those being the issues around the STAR database. The STAR database collects data about social services and benefits provided by municipalities. Stakeholder interviews highlighted that data entry is currently not uniform – the same service can be named and coded differently between the municipalities. The current level of data availability and quality effectively prevents an integration (and subsequent analysis) of the respective social services provided at the municipality level. A clear coding pattern which is uniform between the municipalities is necessary to be able to use the STAR data. The proof of concept analysis showed some issues pertaining to information regarding dates across almost all data sources. Minor issues were also observed concerning the coding schemes of the different data sources, e.g., use of different separators (like comma or period) for the same purpose, coding of monetary values as either cents or euros. Also, while national registries are connected through the X-Road, enabling secure data exchange in principle, there are no existing X-Road services for a lot of the data elements in the current scope.

## Lessons learned

Three key lessons from the exercise should be highlighted.

1. Consistent stakeholder consultations are crucial for achieving stakeholder buy-in. It was evident throughout the reform initiative that when some key stakeholders did not participate in the core team or steering committee meetings or could not do so with sufficient time to prepare, the urgency of the reform initiative was decreased in these organisations. However, when stakeholders were actively engaged in reform preparation activities, had sufficient time to contribute and had their own needs and ambitions represented in the plans, contributing their time and resources was set as a priority.
2. In the complex field of integrated care, the context and factors influencing reform plans are constantly changing, meaning that detailed plans can quickly become irrelevant. The reform team needed to adopt a “go with the flow” attitude and change and renew plans as changes in the context occurred. Agile prototyping of concepts was used to keep the relevance of the reform. While such an agile approach often meant significant additional workload in the short run, it made the outcome more meaningful and useful in the long run.
3. It is important to note that no change happens without policy support. Throughout the course of the work, it was repeatedly seen that if policy and funding decisions were not made, no progress was possible, even if all stakeholders agreed with the course of action in principle. On the other hand, when policymakers saw the need for change, proposals were quickly integrated into ongoing policy changes, leading to real-life change.

The influence of the COVID-19 pandemic also needs to be noted. With regards to consistent stakeholder consultations, it became evident that personal contact, including contact through physical meetings, matters. As the pandemic brought restrictions to travel and physical meetings, the progress of the reform initiative undoubtedly slowed and it became harder to keep the momentum going. Of course, it was partly due to changing priorities as the pandemic took up a lot of the resources of the reform stakeholders. However, the role of personal contact should still not be underestimated. The possibility of other future crisis needs to be considered when designing the organisation of the data centre.

## Conclusion

As part of the integrated care reform initiative in Estonia, a concept of an integrated data centre was developed and tested in close cooperation with relevant stakeholders. The proof-of-concept exercise showed that, in principle, aggregated data to support integrated care policy could be produced, as initially envisioned. Stakeholder consultations clearly showed that there is demand for such data and with reasonable effort, building an integrated data centre would be feasible, both technically and legally. The components of such a data centre were specified as part of the reform preparation work. To proceed with practical establishment of such a data centre, concrete strategic and financial decisions from the Reform Steering Committee are now needed.

## Additional File

The additional file for this article can be found as follows:

10.5334/ijic.6953.s1 Annex. Includes use cases and detailed requirements emerging from the co-design, table of data sources in the test dataset and full specifications for the data center.
