# Supplementary material for: Drilling for ‘New Oil’ in Care Integration – Co-Production of the Concept and Specification of an Integrated Data Centre for Policy Decision Making, Care Planning, and Research in Estonia
Source: Int J Integr Care. 2023 Mar 29;23(1):17. doi: 10.5334/ijic.6953 (PMC10064888; doi:10.5334/ijic.6953)
Supplement: Annex. — Includes use cases and detailed requirements emerging from the co-design, table of data sources in the test dataset and full specifications for the data center. [file ijic-23-1-6953-s1.pdf]

8. Annex

8.1 Use cases and detailed requirements emerging from the co-design

During the co-design process, use cases and requirements were developed and collated with the stakeholders involved. These are presented here in full.

8.1.1 Use cases

In relation to policy formulation and decision making, more specific purposes for the integrated data centre and the dataset can be seen in **demand analysis** (e.g., for new policy measures, new care and support models, etc.), in **impact assessments** (e.g., for new legislation), and **continuous monitoring and benchmarking**. On the research side, the dataset would allow for epidemiological and health services research, but also for usage in real-life trials (e.g., of innovative interventions).

With a view to the main workstreams of the project, the following – more specific – use cases can be envisaged:

Integrated Care Strategy

- Identification of priority areas for further integration measures, e.g., by population size, financial impact, shortcomings of the current support structure
- Monitoring of active strategy elements, e.g., in terms of population envisaged vs. population enrolled, cost, different types of outcome measures
- Optimisation of resource use
- Contributing to increase transparency about system performance that in turn encourages improvement efforts

Financing & Incentives

- Developing and testing different financing models for integrated care specific to the Estonian context, including sensitivity to high-cost cases, resilience in case of pandemics or other disasters etc.
- Determining payment levels for different types of services and different providers
- Facilitation of the development of clear metrics that are credible to drive, e.g., reimbursement decisions
- Cost controlling

Models of Care & Care Coordination

- Understanding demand and population structures for integrated care pathways, including changes over time
- Modelling new integrated care pathways
- Supporting decision making, e.g., allowing decision makers and providers to assess the performance of integrated care pathways along dimensions such as demand/supply match, outcomes, service provision in line with existing guidelines, etc.
- Identifying key hand-over points in existing care trajectories that would require coordination measures
- Monitoring of existing care coordination measures against guidelines
- Improving proactive service planning and provision, e.g., between state and municipality level services and different service sectors

Measures

- Developing and testing suitable measures for the monitoring and benchmarking of integrated care along the line of the quadruple aim
- Experimental or quasi-experimental evaluation designs for integrated care pathways

8.1.2 Requirements

General requirements

- The data centre is conceived as a combination of human, technical, organisational, and legal elements to ensure its operation and the protection of data. The data centre is also envisaged as a learning system, containing procedures that allow for
  - a regular assessment of its performance;
  - a regular comparison of data demand from policy and other decision makers, care planners, researchers, etc. to the data that is actually being provided; and
  - a continuous process of improvement.
- Specifically, the data centre should be supported in its strategic and operational planning by an advisory board, consisting of citizen representatives, representatives of end-users, as well as representatives of relevant scientific communities. This board can contribute to the continuous development of the data centre and may also support more operational aspects, such as quality assurance, dissemination and communication.
- The data centre should allow for a general concept of "Predict – Model – Measure" in order to support continuous improvement of different parts of the integrated support system. In doing so, emphasis should be on comprehensive data from all sectors, as well as on contextual and structural data that allow for a better understanding of framework conditions.
- Due to the constantly changing data world, the data centre should be designed in a way to be open to future data sources, including distributed or real-world data (e.g., from health apps). This widening of the scope of data to be used will require legal, ethical and scientific oversight.
  - Specifically, the data centre should support the direct linkage of other types of data.
- The data centre should be accessible to external analysts and researchers, with data being made available onsite via a data mart set up for remote access.
- The dataset should be accessible both easily and quickly. Current turn-around cycles for policy indicators were deemed too long, esp. regarding existing procedure for obtaining access to and assembling datasets. Average response times are to be measured in months, although crisis situations such as pandemics or natural disasters may require a faster response time. Based on requirements from the expert workshops, data should ideally be available after no more than 3 months lead time.
  - Specifically, ease and speed of access should be supported by measures such as clearly defined data models (minimal dataset definitions), streamlined application processes (including fast track options for urgent data requests) and transparent rules and regulations.
- As part of an open and transparent approach, the data centre should seek active exchange not only with its professional end-users, but also with the general public.

- Along with the legal basis for its operation (cf. below), the data centre will require a secure and independent financial basis, allowing it to fulfil all its functions. This financial basis should specifically allow for data requests at different cost levels, so that smaller scale analyses with no or low financial backing can also be carried out.
- Implementation planning for the data centre (including cost planning) should take into account both the initial or start-up phase and the continuous operation of the centre. The use cases outlined above will require considerable efforts in terms of the collation of specific requirements, definition of data models, and the production of data outputs during the start-up phase. Efforts for continuous operation will largely depend on the actual number of analytical tasks running in parallel.

Legal and regulatory requirements

- The operation of the data centre requires a specific and unambiguous legal framework. This legal basis should be flexible enough to support the concept of a learning system, as described above.
    - This concerns in particular the legal possibility for the data centre to collect and process pseudonymised, individual level data. All experts interviewed expressed the that anonymised or aggregate data are insufficient.
    - Furthermore, the data centre must have a legal framework to request, collect and process data from all relevant data owner organisations. This concerns specifically the collection of data on the municipality level and the data from service providers.
- Data privacy and data protection need to be ensured in compliance with all relevant legislation and regulations, using a combination of legal, technical, and organisational measures. This includes the protection of the individual's identify, particularly by technically preventing direct re-identification (by name, social security number, or similar) and by organisationally preventing indirect re-identification (e.g., by a combination of identifiable characteristics). A detailed data protection concept and data management plan is required, describing all relevant measures. Compliance with data privacy and protection requirements needs to be mandatory for data delivering agencies, the data centre and its sub-units, as well as for the analysts and end-users using the data.
        - For more detailed information on the legal requirements, we refer to the outcomes of the data sharing workshops included in the final report
- As pseudonymisation does not always guarantee anonymity of the data subjects, several additional measures are to be used to prevent de-pseudonymisation in the sense of re-identifying a person. Firstly, pseudonymisation takes place before linking and no analyst has access to both pseudonymised data and personalised data or the pseudonymisation key. Personal ID-s, names, addresses, and any free text comments will be moved from the dataset to reduce the risk of accidental identification. Also, the sequence of data requests is planned in a way to reduce the delivered dataset to data that are strictly necessary to answer the questions. Other common measures such as data blurring methods (eg., showing the year of service instead of the month, if needed) and agreeing on a minimum number of cases to be included in the dataset (thus having some data outputs only on the national and not on the regional level), may also be applied, where appropriate. These may, however, have an undue impact on the analysability of the data. Furthermore, organisational measures (contracts, legal boundaries) need to be established to prevent analysts from exploiting the possibility of reidentification.

    - .

Scientific and methodological requirements

- The data centre should operate in accordance with good scientific practice and relevant scientific and methodological guidelines. Compliance with scientific requirements should be mandatory for data delivering agencies, the data centre and its sub-units, as well as the analysts and end-users using the data. Relevant examples for scientific guidance include the following:
    - Good Practice Data Linkage (GPD): A Translation of the German Version: https://www.mdpi.com/1660-4601/17/21/7852/htm
    - Development and validation of reporting guidelines for studies involving data linkage: https://onlinelibrary.wiley.com/doi/10.1111/j.1753-6405.2011.00741.x
    - The Reporting of studies Conducted using Observational Routinely-collected health Data (RECORD) Statement: https://www.ncbi.nlm.nih.gov/pmc/articles/PMC4595218/
    - A Consensus German Reporting Standard for Secondary Data Analyses, Version 2 (STROSA-STandardisierte BerichtsROutine für SekundärdatenAnalysen). A German standard similar to RECORD but with a higher degree of adaptation to the German context, particularly with a view to data protection: https://www.thieme-connect.com/products/ejournals/html/10.1055/s-0042-108647#R2015-11-193-0050
    - Guidelines and recommendations for ensuring Good Epidemiological Practice (GEP): a guideline developed by the German Society for Epidemiology: https://link.springer.com/article/10.1007/s10654-019-00500-x
- There should be strong commitment to scientific accuracy and adherence to scientific theories and practices. Specific mentioning was made of algorithms for the analysis of social data that need to be in accordance with social theories.
- As mentioned above, the advisory board should contain representatives from the scientific community to provide respective oversight.

Organisational requirements

- A clear product owner for the data centre needs to be identified and its roles, rights and responsibilities specified vis-à-vis the agencies delivering the data and the people and organisations analysing the data. Both TEHIK and Statistics Estonia were specifically mentioned as potential hosts for the data centre.
- The data centre should be organisationally separate from any agency providing data into the dataset. This includes the data centre’s operation outside any form of scientific or economic competition. Within the data centre, there should be organisational boundaries or sub-entities (the trust centre, the data warehouse and the data mart) with clearly defined responsibilities. These sub-entities can potentially also be hosted at separate organisations.
- There needs to be a clear set of rules and conditions for accessing the data, including whether the data can only be analysed within the data centre or also on-site at the analyst’s, under what conditions the data can be analysed, etc. These rules and conditions should be communicated transparently at all times, e.g., through the data centre’s website.

- Similarly, the data in the data centre should be updated regularly in order to maximise usefulness. The general view was that (except for crisis situations) anything approaching real-time data delivery would probably not be necessary. Views about updating frequency ranged from intervals of 1 month to 6 months. Update cycles might also vary depending on how labour intensive it is to update a data source (e.g., for source systems where data is not machine readable).
- Quality of the data is a key concern. On the one hand, the quality of the data and the data management processes should always be checked and improved, on the other hand, a clear view of the shortcomings of the data should exist and be communicated with every analysis. The experience of the people working with different source data will be very important for this.
- Users and analysts of the data should be able to receive support from the data centre for all steps of the data flow, from initial data requests to the analysis of the data. This can take the form of information and training material provided through the website of the data centre, but also regular workshops, one-on-one support, etc.

Human resources need to be considered and should not be underestimated. For the integrated data centre and its applications to be successfully implemented, there needs to be sufficient amount of dedicated personnel, with required skills and competencies, included in both the establishment of the integrated data centre and operating the day-to-day work of both the centre and its applications. Capacity-building is needed among the data owners, data analysts, policymakers and other end users alike. The workforce involved needs to be sufficiently sized and upskilled. There is also potential in creating a kind of learning community around the centre where centre staff and data users can exchange experience.

8.2 Table of data sources in the test dataset

Table 1. Data sources in the test dataset

DATA OWNER	DATA SOURCE	TYPE OF DATA	DATA FIELD
**MINISTRY OF THE INTERIOR**	Population Registry	General data	Personal identifier, Place of residence, Date of birth, Date of death, Gender
**HEALTH INSURANCE FUND**	HIF database	Context data	Insurance status
	HIF claims data	Context data	Diagnosis
	HIF claims data	Healthcare service data	Name of service, Beginning of service, End of service, Quantity of service delivered, Price, Measure of quantity
	HIF claims data	Healthcare benefit	Name of benefit, Date of payment, Quantity of benefit
**MINISTRY OF EDUCATION AND RESEARCH**	EHIS	Context data	Educational attainment level, Status of being currently in education
	EHIS	Context data	Educational special need, Educational special need classification

**SOCIAL INSURANCE BOARD**	AVE	Assessment data. Medical aid	Time of issuing the medical aid referral
	AVE	State level social service data	Name of technical aid, Beginning of service, End of service, Price, Quantity of service delivered, Measure of quantity
	SKAIS	Context data	Disability. Time of assessment, Disability. Validity of status, Severity of disability, Type of disability
	SKAIS	Assessment data. Rehabilitation, Assessment data. Special care	Time of assessment, Result of assessment. Is the service needed?, Basis for service provision
	SKAIS	State level social service data	Name of service, Beginning of service, End of service, Price, Quantity of service delivered, Measure of quantity
	SKAIS	State benefit/pension	Name of benefit/pension, Date of payment, Quantity of benefit, Name of benefit, Date of payment, Quantity of benefit
	STAR	Assessment data. Local municipality needs assessment	Is there a case plan?
	STAR	Local municipality level social service data	Name of service, Beginning of service, End of service, Price, Quantity of service delivered, Measure of quantity
**TAX AND CUSTOMS BOARD**	TÖR	Context data	Employment status
	TÖR	Context data	Salary
**UNEMPLOYMENT INSURANCE FUND**	REDIS	Assessment data. Rehabilitation	Time of assessment, Result of assessment. Is the service needed?
	REDIS	Employment service data	Name of service, Beginning of service, End of service, Price, Quantity of service delivered, Measure of quantity
	TETRIS	Employment benefit	Name of benefit, Date of payment, Quantity of benefit

**MINISTRY OF EDUCATION AND RESEARCH**	EHIS	Assessment data. Educational special needs assessment	Time of assessment, Result of the assessment. Is a service needed?, Which service is needed?
	EHIS	Educational support service data	Name of service, Beginning of service, End of service, Price, Quantity of service delivered, Measure of quantity
**TEHIK**	TIS	Referral data	Time of referral
**UNEMPLOYMENT INSURANCE FUND**	TETRIS	Context data	Work ability. Time of assessment, Work ability. Validity of status, Work ability. Assessment result
**MUNICIPALITY DATA**	Variable	Assessment data. Local municipality needs assessment	Time of assessment, Result of assessment. Is a service needed?, Which service is needed?
	Variable	Local municipality level social service data	Name of service, Beginning of service, End of service, Price, Quantity of service delivered, Measure of quantity

8.3 Full data centre specs

8.3.1 Premises

The architecture and data flow for the dataset and data centre presented here are based on the following premises.

- The approach describes what can be considered “Version 1” of the integrated data centre, specified to fulfil specific functions and deliver specific data products. In keeping with the concept of the learning socio-technical system approach described in the considerations above, these premises are subject to change.
- The data centre supports both one-off requests for analysis and continuous analytic tasks, corresponding to two types of data products to be supported (albeit with the same data flow):
    - analytic reports, relating to one-off analysis and based on specific data requests
    - dashboards of regularly updated data on given subjects and based on specific data requirements
- This first version of the data centre is not based on a permanently assembled centralised dataset, but rather on individual data requests from the delivering agencies, directly linked to each analytic task (reports of dashboards).
    - This implies that the process of learning and continuous improvement of the data centre is limited to the methods and procedures surrounding data

delivery, data preparation, quality assurance and analysis, rather than including a continuously improving dataset itself.

- This approach should be monitored both in terms of its effectiveness and its efficiency, depending also on the number of data requests to be handled within a specified time frame.

8.3.2 Data flow diagram

Table 2. Data flow of the integrated data set

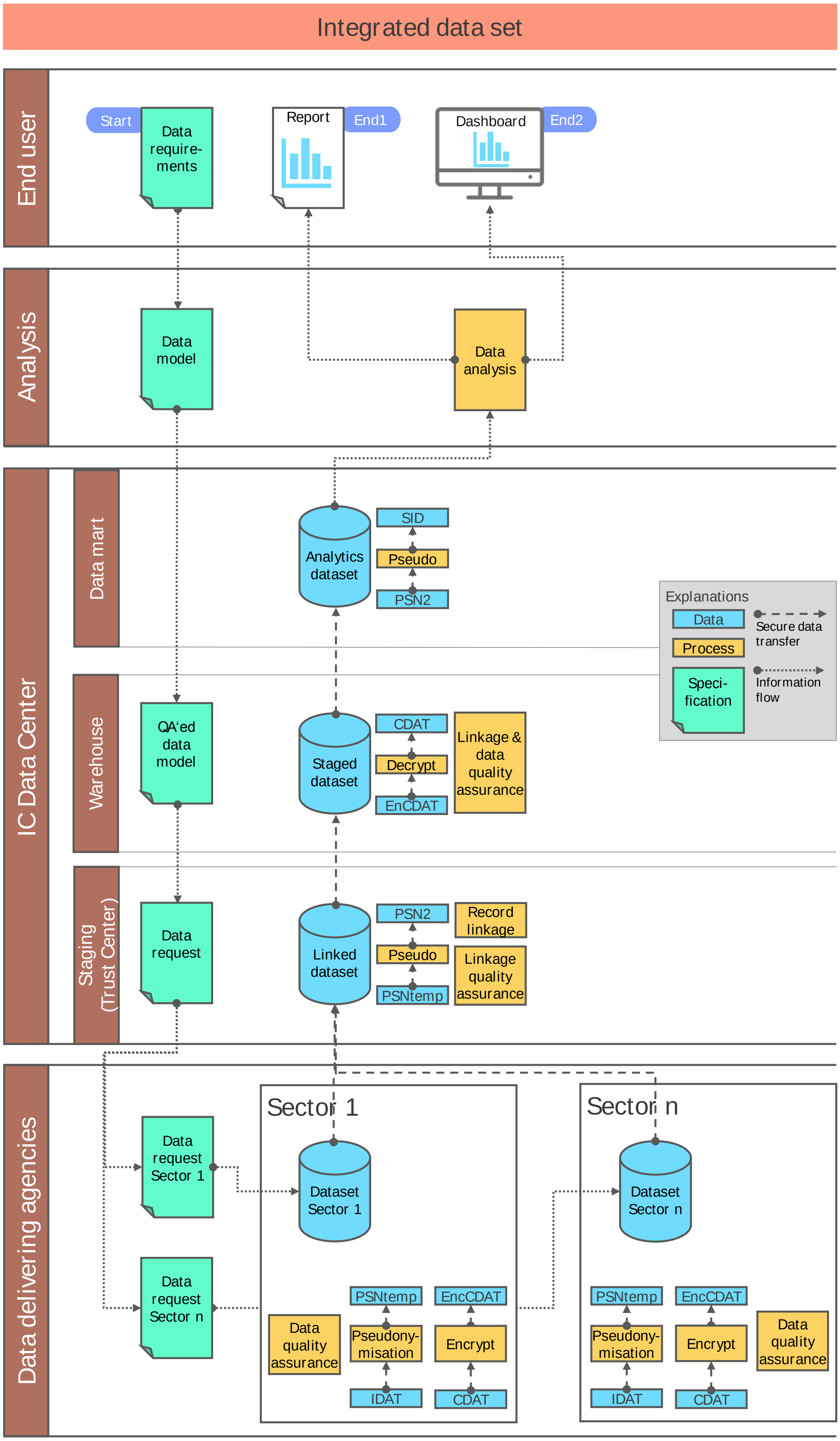

Integrated data set
End user
Start
Data require-ments
Report
End1
Dashboard
End2
Analysis
Data model
Data analysis
IC Data Center
Data mart
Analytics dataset
SID
Pseudo
PSN2
Explanations
Data
Process
Speci-fication
Secure data transfer
Information flow
Warehouse
QA'ed data model
Staged dataset
CDAT
Decrypt
EnCDAT
Linkage & data quality assurance
Staging (Trust Center)
Data request
Linked dataset
PSN2
Pseudo
PSNtemp
Record linkage
Linkage quality assurance
Data delivering agencies
Data request Sector 1
Data request Sector n
Sector 1
Dataset Sector 1
Data quality assurance
PSNtemp
Pseudony-misation
IDAT
EncCDAT
Encrypt
CDAT
Sector n
Dataset Sector n
PSNtemp
Pseudony-misation
IDAT
EncCDAT
Encrypt
CDAT
Data quality assurance

Description of key elements

1) **Start:** Data flow starts on the side of the end-user and is identical for both data products: the one-off data request for a report and the request for data for a continuous dashboard. The specific data requirements for the data product are specified by the end-user.

2) These requirements are then translated into a specific data model by the analyst. As a minimum requirement, this general data model contains a list of data items (indicators, table columns), definitions of data formats, as well as requirements for the selection of the population (age limits, geographical limits etc.). This data model needs to comply with the overall structure of the available data. To support this, the data centre may provide a minimal dataset definition describing all available data profiles. The data model also contains justifications for all data being requested that are specifically linked to the analytic purpose to be served by the data product. As mentioned in the requirements above, the collation of data requirements and the definition of the data model need to be sufficiently resourced.

3) The data model is sent to the data centre and checked for quality, esp. against the requirements of the minimal dataset.

4) The data model is transferred into a data request that is split into different sectors or data delivering agencies from which data is being requested. These sector-specific data requests are transmitted to the data delivering agencies by the data centre.

5) Each data delivering agency selects data based on the data request from its own source system or systems. The dataset is divided into the data used for the identification of the individual (IDAT) and other data referred to as content-related data (CDAT). The IDAT needs to consist of a unique or bijective identifier, allowing for neither synonyms nor homonyms. In case it fulfils this requirement to a sufficient degree, the personal identifier provided in the population register of the Ministry of the Interior is proposed to be used for this purpose.

6) The IDAT undergoes initial pseudonymisation by the delivering agency, using a salted hash function preventing depseudonymisation. The employed algorithm and key need to be identical for all data delivering agencies to allow for direct linkage of each record across sectors. The result is a temporary pseudonym (PSNtemp) attached to each record of CDAT. The CDAT element of each record is encrypted, using a key that is known to the individual data delivering agency and the data warehouse in the data centre but not to the trust centre. This ensures that the trust centre only has access to the data needed for linking records (i.e., the IDAT). The resulting encrypted content data are referred to as the EncCDAT. The combination of PSNtemp and EncCDAT is the specific dataset from each sector / agency relating to the data product. The datasets are delivered to the trust centre within the data centre via secure means.

7) The trust centre within the data centre receives the datasets corresponding to the specific data request and data product from each agency. The trust centre rehashes the PSNtemp to a new PSN2, using an algorithm and key known only to the trust centre. This ensures that the data passed on to the data warehouse cannot be linked back to the original records at the delivering agencies. The trust centre then carries out record linkage and assures the quality of the linkage. The linked specific dataset is then transferred into the data warehouse.

8) In the data warehouse, the EncCDAT are decrypted to CDAT. Further quality assurance regarding the linkage and the data is carried out. This includes compliance with the requirements of the specific data model but also basic plausibility checks, such as frequency counts per sector, per time unit under observation, population characteristics,

etc. Depending on the outcome of quality assurance, additional or updated data deliveries may be necessary, which will follow the same data flow.

9) The staged dataset is then transferred into the data mart where it will eventually be accessed remotely by the analyst. Before this, the PSN2 are rehashed a second time into a study identifier that is specific to the analytics dataset and the data product to be created (SID), thus preventing analysts from linking to datasets created for different data products than their own.

10) **End:** After the analytics dataset is prepared, the analyst receives remote access to the specific dataset in the data mart for the time of the analysis. The data mart allows the exporting of analytic results to be used in reports (End1) or dashboards (End2).

11) After concluding the analytic work, access is revoked and the analytic dataset along with the analytic code is archived by the data centre. Archived datasets are maintained for a pre-defined period of time in order to allow for re-analysis at a later stage, e.g., in case of further inquiries or challenges to results. In cases where a re-analysis becomes necessary, the analytic dataset is re-instated at the data mart.
